# RASAL3 Is a Putative RasGAP Modulating Inflammatory Response by Neutrophils

**DOI:** 10.3389/fimmu.2021.744300

**Published:** 2021-10-27

**Authors:** Suguru Saito, Duo-Yao Cao, Aaron R. Victor, Zhenzi Peng, Hui-Ya Wu, Derick Okwan-Duodu

**Affiliations:** ^1^ Bio-fluid Biomarker Center, Graduate School of Medical and Dental Sciences, Niigata University, Niigata, Japan; ^2^ Department of Biomedical Sciences, Cedars-Sinai Medical Center, Los Angeles, CA, United States; ^3^ Division of Virology, Department of Immunology and Infection, School of Medicine, Jichi Medical University, Shimotsuke, Japan; ^4^ Department of Pathology, Cedars-Sinai Medical Center, Los Angeles, CA, United States; ^5^ College of Animal Science and Technology, Northwest A&F University, Shaanxi, China; ^6^ College of Health Science, Trans World University, Douliu, Taiwan

**Keywords:** neutrophil, acute inflammation, RasGAP, Ras signal pathway, lipopolysaccharide (LPS), sepsis, RASAL3

## Abstract

As first responder cells in host defense, neutrophils must be carefully regulated to prevent collateral tissue injury. However, the intracellular events that titrate the neutrophil’s response to inflammatory stimuli remain poorly understood. As a molecular switch, Ras activity is tightly regulated by Ras GTPase activating proteins (RasGAP) to maintain cellular active-inactive states. Here, we show that RASAL3, a RasGAP, is highly expressed in neutrophils and that its expression is upregulated by exogenous stimuli in neutrophils. RASAL3 deficiency triggers augmented neutrophil responses and enhanced immune activation in acute inflammatory conditions. Consequently, mice lacking RASAL3 (RASAL3-KO) demonstrate accelerated mortality in a septic shock model *via* induction of severe organ damage and hyperinflammatory response. The excessive neutrophilic hyperinflammation and increased mortality were recapitulated in a mouse model of sickle cell disease, which we found to have low neutrophil RASAL3 expression upon LPS activation. Thus, RASAL3 functions as a RasGAP that negatively regulates the cellular activity of neutrophils to modulate the inflammatory response. These results demonstrate that RASAL3 could serve as a therapeutic target to regulate excessive inflammation in sepsis and many inflammatory disease states.

## Introduction

Ras signaling is an indispensable biological process that shapes cell proliferation, differentiation, and activation, allowing cells to respond to environmental cues and maintain homeostasis (1, 2). In response to external stimuli, Ras is converted from its inactive form (Ras-GDP) to its active form (Ras-GTP) by guanine nucleotide exchange factors (GEF) (3). Active Ras-GTP then transmits the external signal *via* the mitogen-activated protein kinase (MAPK) pathway comprising Raf, mitogen-activated protein kinase kinase 1 (MEK), and extracellular signal-regulated kinase (Erk) (4). Ras signaling also activates other factors such as Akt and p38MAPK (5). While activation of the Ras signaling pathway is critical for cellular response to environmental cues, inactivation is required for the maintenance of homeostasis. Indeed, failure to suppress overactivation of Ras signaling leads to dysfunction and disease, including excess inflammation and tumorigenesis (3, 6).

The Ras protein has endogenous GTPase activity which hydrolyzes GTP to GDP. However, this process is relatively slow. Thus, another class of factors, so-called Ras GTPase activating proteins (RasGAPs), are required to accelerate GTP hydrolysis (7). The functional failure of RasGAPs is strongly linked with tumorigenesis. For instance, Von Ricknlinghaysen’s syndrome is cause by mutation of neurofibromin 1 (NF1), perhaps the most studied RasGAP, which drives tumors in various tissues (4). Other RasGAPs—RASAL1, RASAL2 and DAB2IP—are also closely associated with various tumors, further supporting the critical role of RasGAPs as tumor suppressors (8–10). However, the direct of role of RasGAPs in regulating immune cell function is incompletely described.

Another RasGAP, RASAL3, is expressed in many tissue types including lung, kidney, adipose, and brain, but is notably highly expressed in hematopoietic tissues including bone marrow, lymph node and peripheral blood (11). Specifically, RASAL3 is expressed in several hematopoietic cell types, including T-cells, B-cells, and NKT cells, where it plays various roles (11–14). While Ras signaling generally modulates neutrophil function (15), the specific role of RASAL3 in neutrophils is not well-characterized. Thus, we sought to investigate the role of RASAL3 in neutrophils and determine how it may influence systemic inflammation.

In this study, we show that RASAL3 is highly expressed in neutrophils and functions as a RasGAP. We find that RASAL3 limits excessive inflammatory activation, and its deficiency leads to increased septic shock mortality from hyperactive inflammation.

## Materials and Methods

### Mice

C57BL/6J mice were purchased from the Jackson Laboratory (Bar Harbor, ME, USA). RASAL3-deficient mice (RASAL3-KO) were created as previously reported (11). Mice were backcrossed onto C57BL6/J background for at least ten generations. All mice were bread under specific pathogen-free conditions. Female mice between 8 and 12 weeks of age were used for each experiment. The humanized Townes sickle cell mice (hα/hα::βA/βS, hα/hα::-383 γ-βA/-1400 γ-βS) were obtained from Jackson Laboratory (Jax #013071) (16). Colonies were established and bred in house at the Cedars-Sinai Medical Center animal facility. Female homozygous sickle cell (SS) and heterozygous littermates (AS) were used for experiments between 8 and 12 weeks of age.

For the induction of endotoxin shock, mice received LPS intraperitoneal (i.p.) injection (5 mg/kg). After 24 h, neutrophils in the surviving mice were analyzed by flow cytometry. The survival rate was monitored up to 80 h of post i.p. injection. All the experiments were approved by the animal care and use committee of Jichi Medical University (Protocol No.; 20036-01, 20037-01), Northwest A&F University (Protocol No.; 15-10-874) and Cedars Sinai Medical Center (Protocol No.; 8780).

### Reagents and Antibodies

Lipopolysaccharide (LPS) from Escherichia coli O111:B4 (Sigma-Aldrich, St. Louis, MO, USA) was used. Phorbol 12-myristate 13-acetate (PMA), ionomycin, bovine serum albumin (BSA), anti-BSA rabbit IgG and anti-RASAL3 rabbit IgG were also purchased from Sigma-Aldrich. SYTOXRed, DAPI (4’,6-diamidino-2-phenylindole) and CellROX™Green were purchased from Thermo Fisher Scientific (Waltham, MA, USA). A Cytofix/Cytoperm kit with GolgiStop™ was purchased from BD Bioscience (Franklin Lakes, NJ, USA). Anti-CD45 (30-F11), anti-CD11b (M1/70), anti-Ly6G (1A8), anti-CD16/CD32 (2.4G2) (93) and PE donkey anti-rabbit IgG (Poly4064) were all purchased from Biolegend (San Diego, CA, USA). The isotype-matched control for each antibody was purchased from the same manufacturers.

### Bacteria Culture

Frozen methicillin‐resistant *Staphylococcus aureus* stock was purchased from the American Type Culture Collection (ATCC) (Manassas, VA, USA). The bacteria were thawed on ice, and then transferred to a tryptic soy broth (TSB; BD Bioscience, Franklin Lakes, NJ, USA) and cultured at 37°C for 18 h with shaking. The CFU was calculated in each culture. Heat-killed *S. aureus* (HK-SA) was prepared by heating at 95°C for 30 min. The heated *S. aureus* suspension was centrifuged at 10,000 g for 1 min, and the cell pellet was resuspended in PBS.

### Mouse Primary Cell Preparation

Bone marrow (BM) cells were obtained from the tibia and femur as previously reported (17). Briefly, the cells were flushed out from the tibia and femur using a 10 mL syringe with a 27 G needle containing cell culture medium. The cell suspension was filtered through a 70 μM cell strainer and washed once with cell culture medium. The cells were then treated with 1xRBC lysis buffer at room temperature (RT) for 10 min and washed twice with cell culture medium before use.

Splenocytes were prepared from spleen by following a method described in a previous report (18). Briefly, the freshly extracted spleen was crushed and passed through a 70 μM cell strainer with cell culture medium (RPMI 1640 supplemented with 10% fetal bovine serum (FBS), 100 U/mL penicillin, and 100 mg/mL streptomycin. The cells were treated with 1xRBC lysis buffer at RT for 10 min, washed twice with culture medium, and used as splenocytes.

Blood leukocytes were prepared from 100 μL of peripheral blood. The blood sample was diluted with 1xRBC lysis buffer and was incubated at RT for 10 min. After washing twice with PBS/2% FBS, the cells were used as peripheral blood leukocytes.

Liver and kidney mononuclear cells (MNCs) were prepared by following a method described in a previous report (19). Briefly, the organs were finely minced in a dish in cell culture medium, and then further crushed on a 70 μM cell strainer. After washing with culture media, the MNCs were separated by percoll gradient. Each cell population was isolated using magnetic beads separation methodology. Neutrophils were isolated from BM cells, splenocytes, peripheral blood leukocytes mixture and MNCs mixture using an EasySep™ Mouse Neutrophil Enrichment Kit (STEMCELL TECHNOLOGIES, Vancouver, BC, Canada). Monocytes were isolated from BM cells using an EasySep™ Mouse Monocyte Isolation Kit (STEMCELL TECHNOLOGIES). Splenic CD4+ T cells or CD8+ T cells were isolated from splenocytes by using a MagniSort™ Mouse CD4 T cell Enrichment Kit (Thermo Fisher Scientific) or MagniSort™ Mouse CD8 T cell Enrichment Kit (Thermo Fisher Scientific), respectively. The procedures for the cell isolation kit were performed in accordance with manufacturer’s recommendations. The cell purity for each target population was confirmed by flow cytometry to be well over 90% of purity.

### Profiling of RASAL3 mRNA Expression by Real-Time PCR

To analyze RASAL3 mRNA expression, each lineage of immune cells, hepatocyte and tissue-specific neutrophils were prepared as described. For induction of RASAL3, bone marrow-isolated neutrophils (5 x 10^6^) were seeded on 12 well plate in cell culture medium. The cells were cultured with each stimulator (PMA; 500 ng/mL, Ionomycin; 1 µg/mL, LPS; 1 µg/mL, immune complex (IC); formed with BSA 1 µg + anti-BSA rabbit IgG 10 µg) and heat kill staphylococcus aureus; 1 x 10^6^ colony forming units) at 37°C for 3 h. After stimulation, the cells were harvested and treated with TRIzol RNA Isolation Reagents (Thermo Fisher Scientific) to isolate total RNA. cDNA was synthesized with 500 ng of total RNA using the PrimeScript RT reagent kit (TaKaRa, Tokyo, Japan). The expression level of RASAL3 mRNA was quantified by TB green system (TaKaRa) and Thermal Cycler Dice (TaKaRa). GAPDH mRNA expression was used as an internal control. The following primers were used to specifically amplify the target genes. For *Rasal3*: forward; 5’-ACTGCGAGACACGCTGTGAGGA-3’and reverse; 5’-TGCTTCACGCCAACTTGAGAAC-3’, for *Gapdh*: forward; 5-TGTGTCCGTCGTGGATCTGA-3 and reverse; 5’-TTGCTGTTGAAGTCGCAGGAG-3’. All procedures were performed in accordance with product manual and recommendations.

### Cytokine, ROS and Extracellular Trap (NET) Production Assay in Neutrophils

For cytokine production assay, neutrophils (1 x 10^6^) were seeded on a 96-well plate in cell culture medium. The cells were treated with LPS (1 μg/mL) or vehicle control at 37°C overnight. The plate was gently spun, and supernatant was harvested and stored at -80°C until analysis. IL-1β, IL-6 and TNF-α were measured by ELISA.

For ROS production assay, neutrophils (1 x 10^6^) were transferred in 1.5 mL tube, and the cells were treated with LPS (1 μg/mL) or vehicle control at 37°C for 30 min in cell culture medium containing CellROX-Green (Thermo Fisher Scientific). After incubation, the cells were immediately analyzed by flow cytometry. The mean fluorescence intensity (MFI) of ROS signal in CD11b+Ly6G+ gate was detected in the analysis.

For neutrophil extracellular trap (NET) assay, we followed standard flow cytometric approach to measure extracellular DNA (20). Briefly, isolated neutrophils were stimulated with LPS (1 μg/mL) or vehicle control at 37°C for 30 min. Cells were fixed by adding 100 μl of 4% paraformaldehyde (PFA; EMS, PA, US) for 15 min at RT. Neutrophils were stained with 0.1 µM of the plasma membrane-impermeable DNA-binding dye, SYTOXRed and 0.3 nM DAPI (Thermo Fisher Scientific, MA, US). After washing, cells were immediately analyzed by flow cytometry. The percentage of Ly6G/CD11b cells showing double positivity for DAPI and SYTOXRed were quantified as cells undergoing NETosis.

### Flow Cytometry

Cell surface and intracellular markers were analyzed by flow cytometers (FACSCant and LSR-II; BD Biosciences, Franklin Lakes, NJ, USA) with the fluorochrome-conjugated monoclonal antibodies described in reagents and antibodies. The cells were initially incubated with FcR blocker (anti-CD16/32; 2.4G2) at 4°C for 10 min. For surface marker staining, the cells were incubated with antibody at 4°C for 30 min. For intracellular staining for RASAL3, samples were treated as above for surface staining. Then, cells were fixed and permeabilized at 4°C for 20 min using a Cytofix/CytoPerm Kit (BD bioscience, Franklin Lakes, NJ, USA). Cell were incubated with the RASAL3 antibody at 4°C for 30 min followed by secondary antibody. Cells were washed and analyzed immediately. For intracellular cytokine staining, the leukocytes were incubated in cell culture medium in the presence of brefeldin A (10 µg/mL). The sample was firstly stained for extracellular markers, then fixed and permeabilized with Cytofix/CytoPerm buffer as described, followed by staining of intracellular cytokine at 4°C for 20 min. The target population was detected by following a gating strategy indicated in **Figure S1A**. All data were analyzed by BD FACS Diva (BD bioscience, Franklin Lakes, NJ, USA) or FlowJo (Tree Star; Ashland, OR, USA).

### Ras Activation Assay

Ras activation was assessed by detecting Ras-GTP in the cells by using a Ras Pull-Down Activation Assay Biochem Kit (Cytoskeleton, DENVER, CO, USA) according to the product manual. Briefly, neutrophils (1 x 10^7^) were treated with lysis buffer, and the lysates were incubated with the Ras-binding domain of Raf-1 (Raf-RBD) coupled to GST beads at RT for 1 h. After washing, the Raf-RBD beads were treated with RIPA buffer (50 mM Tris-HCl, pH 8.0, with 150 mM sodium chloride, 1% NP-40, 0.5% sodium deoxycholate, and 0.1% sodium dodecyl sulfate). After sonication and boiling, Ras-GTP protein, which is pulled-down with Raf-RBD beads, and total Ras in the cell lysate were detected by western blot.

### Western Blot

Cell lysates were diluted with 5× SDS sample buffer (2% SDS, 62.5 mM Tris–HCl (pH 6.8), 10% glycerol, 0.01% bromophenol blue, 50 mM DTT). The cell lysates (20 μg/well) were separated by SDS-PAGE. The proteins were transferred onto a PVDF membrane using Trans-Blot Turbo™ Transfer System (Bio-Rad Laboratories, Hercules, CA, USA). Membranes were incubated with TBS-T (20 mM Tris–HCl (pH 7.6), 150 mM NaCl, 0.1% Tween 20) containing 5% skim milk at room temperature for 1 h, followed by further incubation with the primary antibody. After washing with TBS-T, the membranes were incubated with secondary antibody. The protein bands were visualized using ECL Western Blotting Detection System (GE Healthcare Bioscience).

### Characterization of Signaling Pathway

NF-κB p65, p38 MAPK or Akt were measure by NFĸB p65 (pS536) + NFĸB p65 Total SimpleStep ELISA^®^ (Abcam, Cambridge, UK), p38 MAPK alpha (pT180/Y182 + Total) ELISA (abcam) or Akt (pS473) + total Akt ELISA Kit (abcam), respectively. The neutrophils (1 x 10^6^) were treated with cell extraction buffer, then immediately stored at -80°C. The sample was centrifuged at 15,000 g for 20 min at 4°C, after which the supernatant was collected as protein sample. The sample was used for detection of NF-κBp65, p38 MAPK and Akt. The whole ELISA procedure was performed by following the manufacturer’s recommendations.

### Enzyme-Linked Immunosorbent Assay (ELISA)

*In vivo* cytokines (IL-1β, IL-6 and TNF-α) were measured by ELISA Ready-SET-Go!™ Kit (Thermo Fisher Scientific) for each target. Measurement of serum aspartate transaminase (AST) as surrogate of liver injury was also performed by ELISA (Abcam) by following manufacturer’s recommendations.

### Statistical Analyses

Graphpad Prism (Graphpad Software, La Jolla, Ca) was used for statistical analysis. The Student’s *t* test and ANOVA were used for comparisons between two groups and multiple groups, respectively. Where multiple comparisons are made, Tukey post-hoc test was used. Values of *p < 0.05, **p < 0.01, and ***p < 0.001 were considered as statistically significant.

## Results

### The RasGAP RASAL3 Is Highly Expressed in Neutrophils at Baseline and Is Markedly Increased Upon Cellular Activation

**Figure 1:**
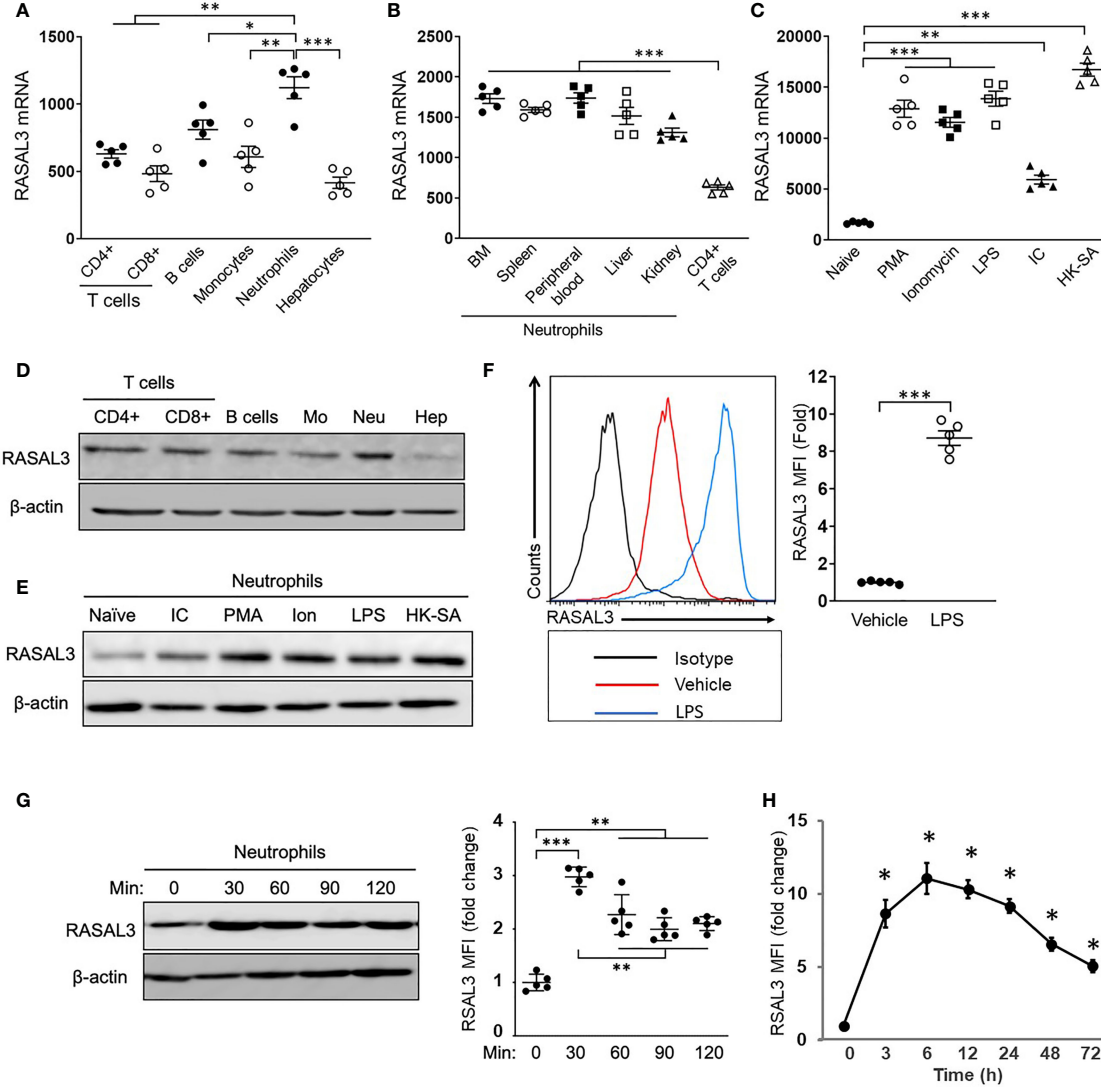
RASAL3 is dominantly expressed in neutrophils and is upregulated by cellular activation. **(A, B)** The characterization of RASAL3 mRNA expression (relative to GAPDH). Total RNA was isolated from each cell population, and RASAL3 mRNA expression was measured by real-time PCR. Basal expression level of RASAL3 mRNA in splenic T cells (CD4+ T, CD8+ T), bone marrow (BM) monocytes, BM neutrophils and hepatocytes **(A)**, **(B)** A comparison of RASAL3 mRNA expression in neutrophils isolated from different tissues (from BM, spleen, peripheral blood, live, kidney). Splenic CD4+ T cells were used as control. **(C)** RASAL3 mRNA expression in stimulated neutrophils. BM neutrophils were stimulated with indicated agonists at 37°C for 3 h: PMA (500 ng/mL), Ionomycin (1 µg/mL), Lipopolysaccharide (LPS) (1 µg/mL), polyinosinic-polycytidylic acid (IC) (BSA 1 µg + anti-BSA mouse IgG 10 µg) and heat killed methicillin resistant S. aureus, strain USA400 (10^6^ CFU/mL). PBS was used as vehicle control. **(D)** RASAL3 protein level in neutrophil populations as determined by Western blotting. Representative blots are shown from cells obtained as described in **(A)**. **(E)** RASAL3 protein expression in bone marrow isolated neutrophils at baseline (naïve) and upon stimulation with various stimuli for 3 h as described in **(C)**. **(F)** RASAL3 protein expression as determined by intracellular staining with flow cytometry analysis. Bone marrow purified cells were stimulated with vehicle or LPS. After 3 h, cells were harvested, washed and stained intracellularly for RASAL3 expression. Data are shown as mean fluorescence intensity (MFI) of expression. **(G, H)** Time-course of RASAL3 protein expression by neutrophils after stimulation with LPS. In **(G)**, neutrophils were stimulated for 30, 60, 90 and 120 minutes. At indicated times, cells were collected and subjected to Western blotting. Protein expression is indicated relative to baseline (unstimulated). In **(H)**, WT mice were challenged *in vivo* (i.p) with LPS. At indicated times, peripheral blood was collected, and RASAL3 protein expression was determined by flow cytometry. Data are presented as fold change in MFI relative to time 0 (before stimulation). The data are shown as mean ± SEM. Each experiment was performed at least two independent times with identical results. n = 5. Each symbol represents independent measurement from one mouse. Values of *p < 0.05, **p < 0.01 and ***p < 0.001.

While Ras signaling is known to play vitals roles in many cell types, the specific contributions of RasGAPs among immune cell types is incompletely defined. We sought to gain further understanding of the role of RasGAPs in neutrophils. We measured baseline RASAL3 mRNA expression in bone marrow-purified neutrophils and several other cell types (**Figure 1A**). Neutrophils showed significantly higher expression of RASAL3 than hepatocytes and other leukocytes, including T- cells, B- cells, and monocytes. Notably, these other cell types largely showed similar RASAL3 expression, approximately 50% of that seen in neutrophils (**Figure 1A**). Because tissue microenvironment has been shown to affect neutrophil transcriptional phenotype (21), we next sought to determine if tissue of origin played a role in neutrophil RASAL3 expression. Remarkably, neutrophil RASAL3 expression was largely consistent across tissues—bone marrow, spleen, peripheral blood, liver, and kidney (**Figure 1B**). We next investigated RASAL3 expression during inflammatory conditions, using several stimuli. Compared to controls, Phorbol 12-myristate 13-acetate (PMA), ionomycin, lipopolysaccharide (LPS), poly[I:C], and heat-killed *S. aureus* all strongly induced RASAL3 mRNA expression (5- to 15-fold) in bone marrow isolated neutrophils (**Figure 1C**).

Comparative protein expression by Western blotting was used to measure increased RASAL3 expression in neutrophils compared to other cells (**Figure 1D**), as well as elevated neutrophil RASAL3 expression upon activation by various stimuli (**Figure 1E**). We also used intracellular staining by flow cytometry to further confirm elevated RASAL3 protein expression after LPS stimulation of cultured bone marrow neutrophils (**Figure 1F**).

To further understand dynamic RASAL3 expression in neutrophils, we performed time-course analysis of RASAL3 expression *in vitro* and *in vivo* upon LPS challenge. As seen in **Figure 1G**, *in vitro* LPS stimulation of neutrophils rapidly induced RASAL3 protein, peaking at 90 minutes. *In vivo*, intraperitoneal challenge by LPS may be used to simulate acute neutrophil-mediated inflammation leading to septic shock. The flow cytometric gating strategy for neutrophils is shown in **Figure S1A**. Here, we found peak RASAL3 induction in circulating neutrophils between 3-6 h, before significant decline after 48 h (**Figure 1H**). These results show that RASAL3 is increased in neutrophils upon activation, and that RASAL3 may modulate the inflammatory response.

It is currently unknown if other RasGAPs are similarly increased in neutrophils to modulate the inflammatory response. Thus, we assessed expression of other RasGAPs in comparison to RASAL3. mRNA levels of RASAL3 was significantly more increased upon LPS stimulation of bone marrow neutrophils when compared with other RasGAPs (**Figure S1B**). Overall, these results suggest that RASAL3 may be a dominant RasGAP modulator of neutrophil inflammatory response.

### RASAL3 Regulates Cell Activation as a RasGAP Protein in Neutrophils

**Figure 2:**
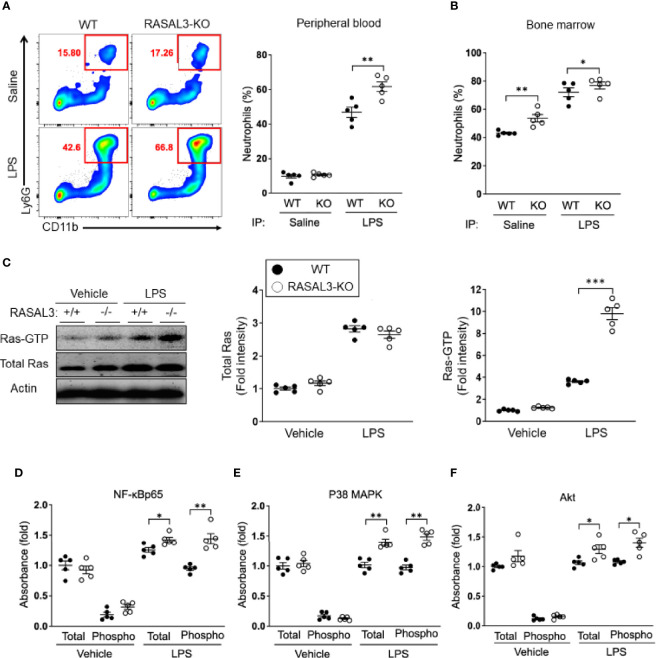
RASAL3 functions as a RasGAP to regulate Ras signaling in neutrophils. **(A, B)** Assessment of neutrophil numbers in RASAL-KO and WT after LPS challenge. Mice received i.p. injection of LPS or saline (vehicle control). After 24 h, peripheral blood and bone marrow was collected, and neutrophil population (Ly6G+CD11b+ cells) were analyzed by flow cytometry. Representative flow cytometric plots are shown, along with percentage of neutrophils in the peripheral blood in **(A)**. Bone marrow neutrophil frequency are indicated in **(B)**. **(C)** Bone marrow (BM) isolated neutrophils were treated with LPS or vehicle control at 37°C for 30 min. Cell lysates were then harvested for Western blot after pull down assays. Active Ras (Ras-GTP) was collected by pull-down. Ras-GTP in the sample and total Ras in whole cell lysate were detected by western blot (WB). A representative image of WB (left) and fold intensities of total Ras (middle) and Ras-GTP (right) in the WB is shown. **(D–F)** Assessment of activation level of downstream inflammatory signaling factors in neutrophils. Both total and phosphorylated levels of NF-κB p65 **(D)**, p38 MAPK **(E)** and Akt **(F)** in cell lysate were detected by ELISA. Data are shown as the mean ± SEM of five samples from one of two independent experiments. Each symbol represents data from one mouse. *p < 0.05, **p < 0.01 and ***p < 0.001.

To characterize the role of RASAL3 during inflammatory challenge *in vivo*, we generated RASAL3 knockout (RASAL3-KO) murine model (11). As expected, the neutrophils of these mice completely lack RASAL3 expression (**Figure S2**). Twenty-four hours following treatment with LPS, we found that the frequency of circulating and bone marrow Ly6G+CD11b+ neutrophils were significantly increased in the RASAL3-KO mice compared to wild-type (WT) mice (**Figures 2A, B**). These data show that RASAL3 modulate the magnitude of neutrophil-mediated acute inflammatory responses and may play a role in driving hyperinflammation during sepsis.

**Figure 3:**
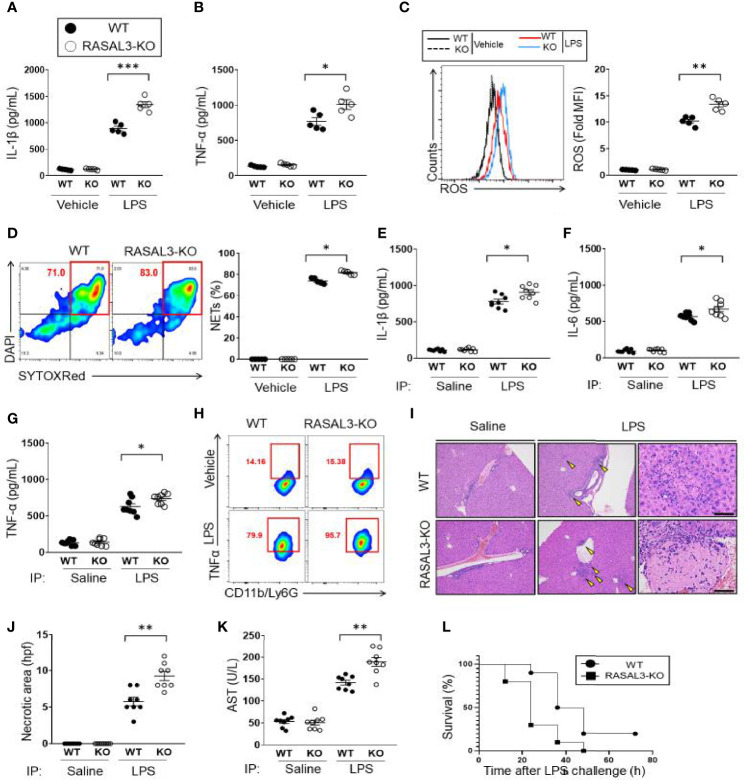
RASAL3-deficiency exaggerates inflammatory response in neutrophils. **(A, B)** Inflammatory cytokine production in neutrophils. Bone marrow isolated neutrophils were incubated with vehicle control or LPS at 37°C for 24 h. The production of IL-1β **(A)** and TNF-α **(B)** were detected by ELISA. **(C, D)** ROS production in neutrophils. BM isolated neutrophils were incubated with vehicle control or LPS at 37°C for 30 min. Flow cytometry was used to determine CellROX-Green assay fluorescence. A representative flow cytometric plot and calculated mean fluorescence intensities (MFI) are shown. **(D)** NET production by BM isolated neutrophils. After treatment with vehicle or LPS, SYTOXRed+DAPI+ cells were quantified as fraction of neutrophils undergoing NETosis. E-H). *In vivo* cytokine production by WT and RASAL3-KO mice. Mice received i.p. injection of LPS or saline (vehicle control). At 12 h, serum was collected, and IL-1β **(E)**, IL-6 **(F)**, TNF-α **(G)** were measured by ELISA. **(H)** Intracellular cytokine (TNF- α) measurement of neutrophils in LPS-challenged WT and RASAL3-KO mice. 6 h after LPS challenge (or control injection), blood was harvested from WT and RASAL3-KO mice, and the proportion of neutrophils (C11b/Ly6G) staining positive intracellular TNF-α were determined by flow cytometry. **(I)** Representative H&E histological analysis of liver necrosis in WT and RASAL3-KO mice in control and LPS challenged mice. **(J)** Quantification of liver necrotic areas per high power field (hpf) are shown for WT and RASAL3-KO mice 12 h after LPS challenge **(K)**. ELISA measurement of serum aspartate aminotransferase (AST) in WT and RASAL3-KO mice 12 h after LPS challenge. **(L)** Kaplan-Meier survival curve analysis for WT and RASAL3-KO mice after LPS challenge (0 through 72 h). Except for L, all data are shown as the mean ± SEM. n=5-10 samples from at least two independent experiment. *p < 0.05, **p < 0.01 and ***p < 0.001.

In a previous report, we found that RASAL3 functioned as a RasGAP in lymphoid cells (11). Thus, we hypothesized that RASAL3 may function similarly in neutrophils. We probed this possibility by performing Ras activation assays in neutrophils. Total RAS was similar between WT and RASAL3-KO without stimulation (vehicle control) and with stimulation (LPS), though stimulation increased total RAS ~3-fold (**Figure 2C**). Under basal conditions, active Ras, which is bound with GTP (Ras-GTP), was slightly increased in bone marrow-derived neutrophils of RASAL3-KO mice compared to those of WT mice (**Figure 2C**). Ras-GTP was greatly increased following LPS stimulation in both WT and RASAL3-KO neutrophils (**Figure 2C**). However, the most striking finding was the fold change in Ras-GTP expression in RASAL3-KO neutrophils, which was significantly greater than that of WT neutrophils upon LPS activation **Figure 3C**, right panel.

We next examined the activation of downstream factors in the Ras signaling pathway, which involves phosphorylation of target genes including NF-ĸB p65, p38 MAPK and Akt (5). The expression levels of both total and phosphorylated NF-ĸB p65, p38 MAPK and Akt were all upregulated in the neutrophils with LPS stimulation (**Figures 2D–F**). As expected, given the upregulation of the active form of Ras, Ras-GTP, in RASAL3-KO neutrophils, these downstream targets were significantly more increased in RASAL3-KO neutrophils (**Figures 2D–F**). These findings support the hypothesis that RASAL3 functions as a RasGAP in neutrophils. Taken together, these activation assays demonstrate that RASAL3-deficiency allows hyperactivation of the Ras signaling pathway under specific stimulatory conditions, such as exposure to exogenous ligands like endotoxin.

### RASAL3-Deficiency Permits an Enhanced Inflammatory Response in Neutrophils

Having established that RASAL3-deficiency allows exaggerated upregulation of Ras-GTP and subsequent induction of downstream intermediates in the Ras signaling pathway, we next sought to determine how this signaling affects neutrophil function. First, we investigated cytokine production. Without stimulation (vehicle control), proinflammatory mediators such as IL-1β and TNF-α showed low baseline expression that was indistinguishable between both WT and RASAL3-KO neutrophils derived from the bone marrow (**Figures 3A, B**). However, under LPS stimulation, production of IL-1β and TNF-α was more strongly upregulated in RASAL3-KO than WT (**Figures 3A, B**). Next, we assessed production of reactive oxygen species (ROS). As with cytokine production, there was low basal expression in both genotypes without stimulation, but robust ROS production following LPS treatment. Again, production was stronger in RASAL3-KO than WT (**Figure 3C**). Moreover, the formation of neutrophil extracellular trap (NET) was also enhanced in RASAL3-KO neutrophils compared to WT after LPS stimulation (**Figure 3D**). Thus, RASAL3 deficiency leads to upregulation of Ras signaling and subsequent augmentation of multiple aspects of the neutrophil inflammatory behavior following activation by exogenous ligand.

### RASAL3-Deficiency Augments Systemic Inflammation and Increases Mortality Rate in Endotoxin-Induced Septic Shock

Next, we investigated how deficiency of RASAL3 affects the systemic inflammatory response and survival in a model of septic shock induced by intraperitoneal injection of LPS. Serum inflammatory cytokine levels showed that LPS strongly induced expression of IL-1β, IL-6, and TNF-α, which were all exaggerated in the RASAL3-KO mice (**Figures 3E–G**). Intracellular cytokine analysis by flow cytometry further confirmed increased number of TNF-α producing circulating neutrophils of RASAL3-KO mice compared to WT. A representative flow cytometric plot 6 h of LPS challenge is indicated in **Figure 3H**. Assessment of neutrophil TNF-α production at other time points are shown in **Figure S3A**. Interestingly, by contrast, cytokine production by other myeloid cells such as peritoneal macrophages and bone marrow derived dendritic cells was indistinguishable between WT and RASAL3-KO (**Figures S3B,C**). Thus, neutrophil deficiency of RASAL3 is the predominant driver of exaggerated inflammatory responses in RASAL3-KO mice.

As the liver is commonly injured in LPS-induced septic shock, we collected histologic section of murine liver to assess for evidence of necrosis. We found evidence of liver necrosis in all mice treated with intraperitoneal LPS injection. Again, this was far more severe in the RASAL3-KO than in WT (**Figures 3I, J**). Indeed, serum aspartate transaminase (AST) which was elevated following LPS injection, was markedly more pronounced in RASAL3-KO than in WT (**Figure 3K**). Consistent with these results, when LPS-injected mice were followed for 72 h, mortality was significantly increased in the RASAL3-KO mice (**Figure 3L**). All RASAL3-KO mice died by 48 h while 40% of WT mice were alive at 48 h; 20% of WT mice survived through 72 h of observation post-injection. Taken together, deficiency of RASAL3 is associated with unregulated production of pro-inflammatory cytokines, increased liver toxicity from hyperinflammatory response, and decreased survival. These data show that RASAL3 plays a significant role in mediating systemic inflammation.

### Hyperinflammation in Sickle Cell Disease Is Driven by Neutrophil RASAL3 Deficiency

Numerous chronic inflammatory disease states lead to increased risk of tissue injury and mortality from sepsis (22, 23). However, the mechanisms by which neutrophils from these conditions promote sepsis mortality remains incompletely understood. We hypothesized that RASAL3 deficiency in neutrophils may contribute to sepsis pathogenesis. Our model of choice to test this hypothesis was sickle cell disease, a hyperinflammatory disease state driven by neutrophil dysfunction (23–25).

**Figure 4:**
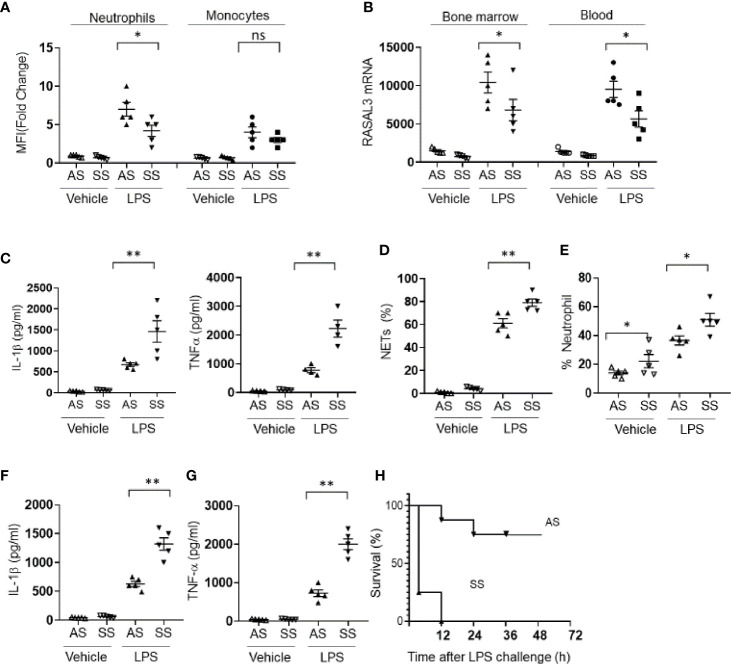
RASAL3 deficiency drives hyperinflammation in sickle cell disease. **(A)** RASAL3 protein expression in BM neutrophils and monocytes from littermate sickle cell Townes (SS) and heterozygotes (AS). 3 h after LPS challenge, mice were sacrificed, and cells were obtained for analysis. Data are represented as fold change in mean fluorescence intensity (MFI) relative to expression levels in unstimulated cells. **(B)** RASAL3 mRNA in bone marrow and circulating blood neutrophils of AS and SS at baseline, and 3 h after LPS challenge. **(C)** Inflammatory cytokine production in neutrophils from AS and SS mice at baseline and after LPS challenge. Bone marrow isolated neutrophils were incubated with vehicle control or LPS at 37°C for 24 h. The production of IL-1β, TNF-α were detected by ELISA. **(D)** NET production was determined by flow cytometric assessment for SYTOXRed+DAPI+ neutrophils after LPS or vehicle challenge. **(E)** Proportion of neutrophils in the circulation of AS and SS at baseline and after i.p. LPS injection (6 h). **(F, G)** Inflammatory cytokine production *in vivo* by AS and SS mice upon challenge with LPS. IL-1β **(F)** and TNF-α **(G)** levels in the blood were measured by ELISA. **(H)** Kaplan-Meier survival curve of AS and SS after LPS challenge *in vivo* (n=10 per group). Except H, data are shown as the mean ± SEM. Each symbol represents one mouse. Each experiment was performed at least two independent times. *p < 0.05 and **p < 0.01. NS, not significant.

We first assessed RASAL3 protein expression in circulating neutrophils and monocytes from Townes mouse model of sickle cell disease. Compared to littermate heterozygotes (AS), sickle cell mice (SS) expressed suppressed levels of neutrophil but not monocyte RASAL3 upon activation by LPS (**Figure 4A**). The decreased neutrophil RASAL3 in SS was confirmed at the mRNA level for both circulating and bone marrow isolated neutrophils (**Figure 4B**). When bone marrow purified neutrophils from SS mice were challenged with LPS, the inflammatory response was markedly exaggerated, with increased production of cytokines such as TNFα, IL-1β (**Figure 4C**), and NETs (**Figure 4D**). Indeed, in response to intraperitoneal challenge with LPS, the frequency of neutrophils were increased significantly in the SS compared to AS, in agreement with exaggerated response (**Figure 4E**). *In vivo* production of proinflammatory cytokines such as IL-1β and TNF-α (**Figures 4F, G**) were markedly increased in the SS after LPS challenge. Unsurprisingly, the mortality rate from LPS-induced sepsis was far more severe in the SS compared to AS (**Figure 4H**). While specific RASAL3 agonists are unfortunately unavailable to determine proof-of-concept reversal of this inflammatory phenotype, the results support that neutrophil RASAL3 deficiency leads to unregulated neutrophil hyperinflammation.

## Discussion

As first response immune cells, neutrophils play pivotal roles in initiating the inflammatory response to various stimuli. The subsequent influx of myelomonocytic cells, which leads to efferocytic clearance of neutrophils in most scenarios, effectively curtails the inflammatory response (26). However, whether and how neutrophils themselves contribute to the regulation of the magnitude of the inflammatory response remains unknown (27). Addressing this knowledge gaps is crucial for the comprehensive understanding of mechanisms to tone down inflammation, the extent of which may lead to collateral tissue damage and mortality, as occurs in sepsis (28).

GTPase activating proteins (RasGAP) regulate cellular active-inactive states. Recently, we reported that RASAL3 functioned as a novel RasGAP in immune cells of the lymphoid lineage (11). In that study, we found that RASAL3 regulated the development and function of natural killer T (NKT) cells. While RASAL3 mediates other lymphocyte differentiation, survival and function (12, 13), myelomonocytic lineage cells also increased RASAL3 expression (11). These findings raised the possibility that RASAL3 may function as a RasGAP in other immune cell types. Here, we demonstrate that RASAL3 plays a significant role in regulating the neutrophil’s contribution to the inflammatory response in the presence of exogenous ligands, both at the cellular level and systemically.

Despite a higher baseline expression level of RASAL3 than other cell types, neutrophils markedly upregulate RASAL3 in response to exogenous ligands. This initial change seems to underpin the role of RASAL3 as a limiting factor in the magnitude of the inflammatory response. Diminished RASAL3 led to exaggerated inflammatory response by neutrophils. Interestingly, macrophages and dendritic cells from RASAL3-deficient mice had similar cytokine production capacity as WT cells, at least upon stimulation with LPS. Thus, it appears that RASAL3 function specifically in neutrophils to initiate the process by which neutrophil-driven inflammatory response is downregulated.

Exogenous ligands such as LPS induce a marked inflammatory response, featuring increased numbers of circulating neutrophils along with enhanced neutrophil effector functions, such as ROS production and NET release. These effects are further increased in the setting of RASAL3 deficiency. Interestingly, these effect sizes are roughly proportional to the differences in phosphorylated Ras pathway signaling intermediates (NF-κBp65, p38 MAPK, and Akt) observed between neutrophils from RASAL3-KO and WT mice. Thus, loss of RASAL3 permits excess production of active Ras (Ras-GTP), which activates its downstream targets and mediates pro-inflammatory effects. These effects were observed at the cellular level and were systemically apparent as well, as evidenced by levels of circulating cytokines, liver damage, and decreased survival in a septic shock model. Finally, in a Townes sickle cell mouse model of hyperinflammation, the exaggerated neutrophils response was associated with reduced neutrophil RASAL3 levels.

As in other immune cell types, RASAL3 deficiency results in increased Ras signaling in neutrophils, evidenced by increased activation (phosphorylation) of downstream intermediates, including Erk. Interestingly, however, while RASAL3 deficiency results in fewer lymphocytes with decreased effector functions (11–13), our present study showed opposite effect of RASAL3 in neutrophils; namely, deficiency leads to an increased number of neutrophils and increased pro-inflammatory cytokine response. The reason for this divergent cellular effect is unclear. Indeed, the pattern of effects observed in RASAL3-KO neutrophils is in line with expectations based on known Ras signaling. Additional study to understand the biological relevance of this discordant effect of RASAL3 in different hematopoietic lineage cells is warranted.

Whether RASAL3 regulates other aspects of inflammatory response by neutrophils, including adhesion, chemotaxis, and communication with other inflammatory cell types, warrant further investigation in future work. Given the abundance of RasGAPs, it is possible that other family members regulate different components of neutrophil biology to lend fine control of these cells. Indeed, other RasGAPs beside RASAL3 may regulate inflammatory response by other innate immune cells. Thus, it remains critical to comprehensively characterize the overall topology of RasGAPs in the control of innate immune responses remains. This effort will benefit from the advent of RASAL3 agonists and antagonists, as well as tissue-specific deletion of RASAL3 and other RasGAPs in animal models. Indeed, the lack of these reagents and techniques represent major limitations of our current work.

Sepsis is a serious clinical challenge with high mortality rate (29). Current therapeutic options involve global targeting of inflammation without specificity for cell type. For instance, corticosteroids, a common agent used in the management of sepsis, not only reduce neutrophilic inflammation, but also may disrupt other immune cellular functions (30). Moreover, corticosteroid use is associated with extensive side effects likely related to their non-specific effects, and its overall effectiveness remains debatable (31). Finer targeting of delineated cell-specific pathways will likely lead to effective regulation of the inflammatory response in sepsis. Our study shows that RASAL3 functions as a brake on the Ras signaling pathway in neutrophils, dynamically moderating the inflammatory cascade in response to external stimuli. The identification and use of RASAL3 agonists, which are yet to be identified at the time of this report, may serve as a novel therapeutic target in sepsis and other neutrophil-driven inflammatory conditions.

## Data Availability Statement

The raw data supporting the conclusions of this article will be made available by the authors, without undue reservation.

## Ethics Statement

The animal study was reviewed and approved by Institutional Animal care and use committee, Cedars-Sinai Medical Center and Jichi Medical University.

## Author Contributions

SS and DO-D conceived the study. SS, AV, D-YC, AV, ZP, and DO-D performed the experiments. SS, AV, and DO-D wrote the manuscript. SS, D-YC, AV, ZZ, H-YW, and DO-D edited the manuscript. SS, HYW, and DO-D: resource and funding (K99HL141638-01A1). All authors contributed to the article and approved the submitted version.

## Funding

This study was funded by the NIH grant number K99HL141638 to author DO-D.

## Conflict of Interest

The authors declare that the research was conducted in the absence of any commercial or financial relationships that could be construed as a potential conflict of interest.

## Publisher’s Note

All claims expressed in this article are solely those of the authors and do not necessarily represent those of their affiliated organizations, or those of the publisher, the editors and the reviewers. Any product that may be evaluated in this article, or claim that may be made by its manufacturer, is not guaranteed or endorsed by the publisher.
