# Supplementary material for: RASAL3 Is a Putative RasGAP Modulating Inflammatory Response by Neutrophils
Source: Front Immunol. 2021 Oct 27;12:744300. doi: 10.3389/fimmu.2021.744300 (PMC8579101; doi:10.3389/fimmu.2021.744300)
Supplement: Supplementary file 1 [file DataSheet_1.docx]

**Supplemental Methods**

*Thioglycolate elicited peritoneal macrophages*

Thioglycolate induction of macrophages was performed as previously described (1). In brief, mice were challenged with 2 ml aged sterile thioglycolate broth. Four days after challenge, mice were euthanized. The peritoneal cavity was lavaged with cold PBS/2% BSA. Cells were plated in tissue culture dishes with complete culture media (RPMI 1640 supplemented with 10% fetal bovine serum (FBS), 100 U/mL penicillin, and 100 mg/mL streptomycin). Adherent cells after 2 h of culture were > 95% F4/80/CD11b positive cells (macrophages). Cells were stimulated with LPS (1 µg/mL) in complete media for 24 h, and cytokine levels were measured by ELISA as previously described.

Bone marrow dendritic cells (DC) were obtained by flushing the femur and tibias as described for isolation of neutrophils. At day 0, 2 × 10^6^ cells were suspended in 10 mL of DC culture medium (cell culture medium containing 20 ng/mL each of recombinant GM-CSF and IL-4), and the cells were seeded on a 100-mm cell culture dish. At day 3, 10 mL of fresh DC culture medium was added to the cultured cells. At days 6 and 8, half of the cultured medium was collected and centrifuged; then, the cell pellets were resuspended into 10 mL of fresh DC culture medium. The cell suspension was put back into the original plate. At day 10, the cells were used for the experiment as naive DC alone or with stimulation with LPS.

**Supplemental Data**


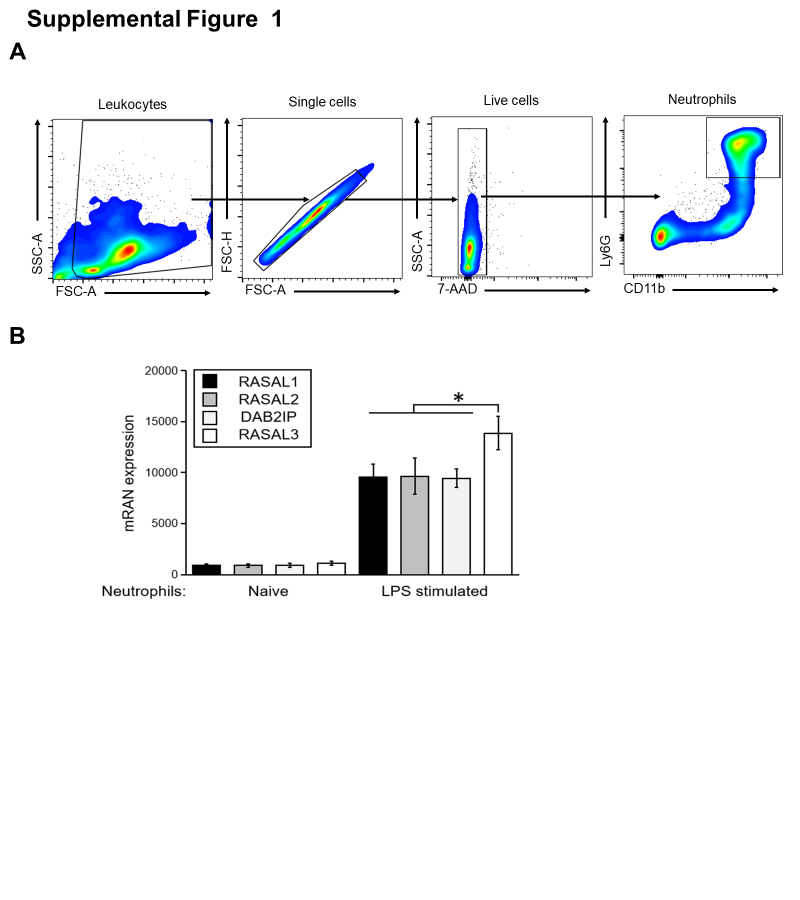


**Supplemental figure 1. Characterization of RASAL family mRNA expression in neutrophils**

A) Gating strategy for the identification of neutrophils by flow cytometry

B) RasGAP mRNA expression in neutrophils. The neutrophils were isolated from bone marrow and then treated with LPS or vehicle control at 37℃ for 3 h. The total RNA was isolated from the samples and subjected to real-time PCR to analyze mRNA expression of RASAL family genes. Data are shown as the mean±SD of five samples. **p*<0.01 was by Mann Whitney U test.


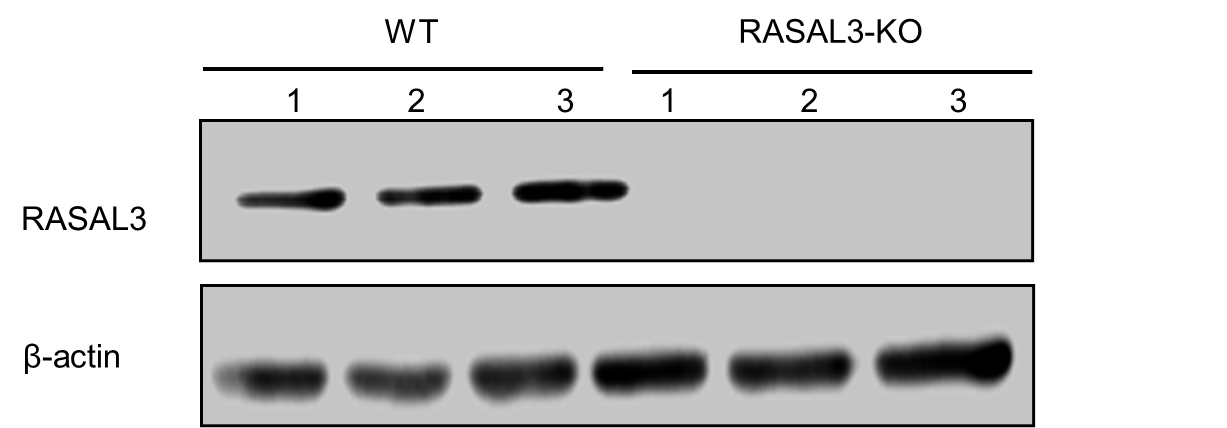


**Supplemental Figure 2.**

RASAL3 protein expression in bone marrow isolated neutrophils isolated from WT and RASAL3-KO mice.


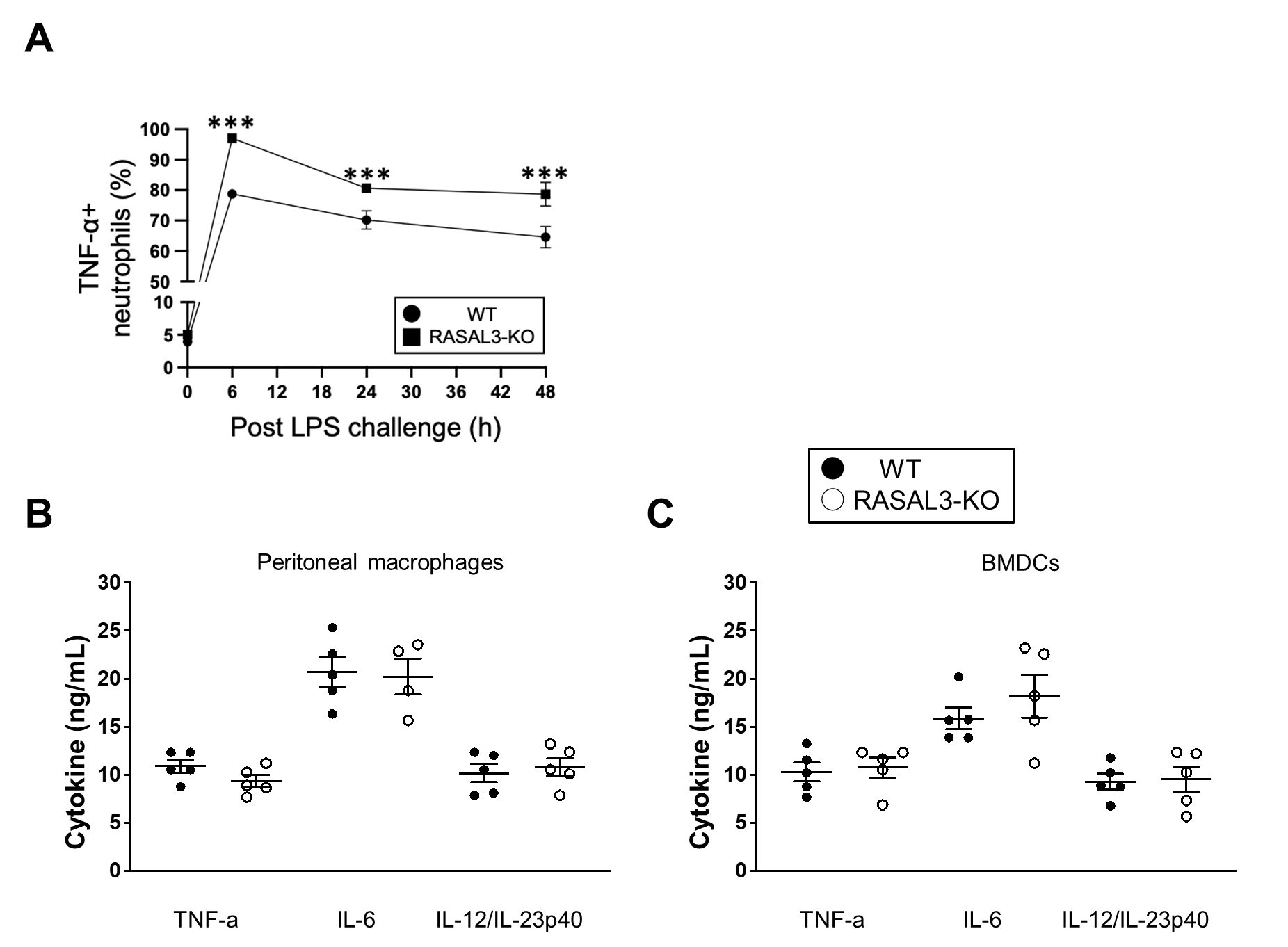


**Supplemental Figure 3.**

A) Time course of TNF-α expression by neutrophils after LPS challenge in vivo. WT and RASAL3-KO mice were challenged with 5 mg/kg LPS. At indicated times, blood was harvested from mice, and Ly6G+CD11b+ neutrophils were stained intracellularly for TNF-α, and the proportion of total neutrophils staining TNF-a are indicated. B-C) Cytokine production by peritoneal macrophages and bone marrow derived dendritic cells from WT and RASAL3-KO mice. Cells were obtained as described in supplementary methods, and stimulated with LPS (1 µg/mL). Cytokines (TNF-α, IL-6, and IL-12) were measured by ELISA. No statistically significant differences were found for any cytokines between WT and RASAL3 KO mice.

**Reference**

1. Cao DY, Spivia WR, Veiras LC, Khan Z, Peng Z, Jones AE, et al. ACE overexpression in myeloid cells increases oxidative metabolism and cellular ATP. *J Biol Chem.* 2020;295(5):1369-84.
